# Correction: Detection and recognition of foreign objects in Pu-erh Sun-dried green tea using an improved YOLOv8 based on deep learning

**DOI:** 10.1371/journal.pone.0321409

**Published:** 2025-03-28

**Authors:** The PLOS One Staff

There are errors in the author affiliations. The correct affiliations are as follows:

Houqiao Wang^1,2^, Xiaoxue Guo^1^, Shihao Zhang^1^, Gongming Li^1^, Qiang Zhao^1^, Zejun Wang^2^

**1** College of Mechanical and Electrical Engineering, Wuhan Donghu University, Wuhan, China, **2** College of Tea Science, Yunnan Agricultural University, Kunming, China

The publisher apologizes for the error.

## References

[1] Wang H, Guo X, Zhang S, Li G, Zhao Q, Wang Z. Detection and recognition of foreign objects in Pu-erh Sun-dried green tea using an improved YOLOv8 based on deep learning. PLoS One. 2025;20(1): e0312112. doi: 10.1371/journal.pone.0312112 39775324 PMC11709275

